# Supplementary material for: Perspectives of frontline health workers on transition from development assistance for health in Ghana: A qualitative study
Source: PLOS Glob Public Health. 2022 Apr 22;2(4):e0000093. doi: 10.1371/journal.pgph.0000093 (PMC10021137; doi:10.1371/journal.pgph.0000093)
Supplement: S1 Text — (DOCX) [file pgph.0000093.s002.docx]

S1 Text

This interview guide is to collect data on the perceptions of health workers on Ghana’s transition from Development Assistance for Health. I will be grateful if you could make time to answer the questions. Every piece of information will be held in absolute confidence. Thank you.

Date……………………………………………………

Interviewer…………………………………………

**INTERVIEW GUIDE**

**Stakeholders awareness and understanding of in-country transitions from external aid to domestic financing for health**

1. What do you understand donor transitions in Ghana to mean?
2. How would you define a successful transition from external aid within the health sector in Ghana?
3. Are you aware Ghana is currently in a state of transition in the health sector?
   1. If **YES**, what is the state of transition in the health sector in Ghana?
   2. Based on your experience at your organization, is there a plan or process in Ghana to support the donor transition?

If **YES**, ask for details and documentation

- 1. Do you think Ghana is adequately prepared for transition? Why?

1. Do you know of any past and future graduation from external aid in the health sector in Ghana?

**Organizational Challenges and Opportunities**

1. Based on your experience at your organization, what challenges did (would) [insert name of organization] face as a result of donor transitions in Ghana?

For ***each challenge mentioned***, ask:

- 1. How did (would) [insert name of organization] address this challenge?
  2. Did (Do) you think your [organization’s] response to the challenge was (will be) successful? Why?

1. What opportunities for improvement did (would) [insert name of organization] have as a result of donor transitions in Ghana?

Ask for details on how they think these challenges did (will) impact health (access to services, outcomes, etc.)

For ***each opportunity mentioned***, ask:

- 1. How did (could) [insert name of organization] take advantage of this opportunity?
  2. Do you think your [organization] was (is) able to take advantage of this opportunity? Why?

1. What lessons, if any, has [organization] learned from its experiences with donor transitions?

**Preferences for policy solutions**

1. Knowing what you know about the challenges and opportunities presented by donor transitions, what do you think is the best way to organize/manage donor transitions at the country level?
   1. When should countries be eligible for graduation from external aid?
   2. When should transition planning start?
   3. How long should the transition process last?
   4. How should countries monitor the donor transition process?
   5. Which stakeholders should be involved in the transition process? May prompt to ask about civil society and representatives from vulnerable populations.
   6. What strategies can ensure that gaps in financing and service delivery are filled by domestic resources?
2. In your opinion, what do you think are the biggest challenges faced by the health sector in Ghana when it experiences donor transitions? How might these challenges impact health (access, delivery, outcomes), especially for vulnerable populations?

For ***each challenge mentioned***, ask:

- 1. How can this challenge be addressed?
  2. Should a country’s ability (or lack thereof) to address this challenge determine if donor aid to the health sector stops or continues? Why?

1. In your opinion, what are the biggest opportunities for the health sector in Ghana when it experiences donor transitions? How might this impact health (access, delivery, outcomes), especially for vulnerable populations?

For ***each opportunity mentioned***, ask:

- 1. What is the best way to take advantage of this opportunity?

1. In your opinion, what are the biggest obstacles to improving health system efficiency during donor transitions?
2. Should donors still provide external aid to the health sector in countries that have graduated from receiving general aid? Why?

Can you recommend other key informants we can interview about donor transitions in Ghana?
