# Supplementary material for: Perspectives of frontline health workers on transition from development assistance for health in Ghana: A qualitative study
Source: PLOS Glob Public Health. 2022 Apr 22;2(4):e0000093. doi: 10.1371/journal.pgph.0000093 (PMC10021137; doi:10.1371/journal.pgph.0000093)
Supplement: Attachment — Submitted filename: Response to comments.docx [file pgph.0000093.s003.docx]

| **S/N** | **COMMENT** | **RESPONSE TO REVIEWER COMMENTS** |
| --- | --- | --- |
| 1  2  3  4  5  6 | **Comments from Editor**  This is an interesting article that will add to the literature on health services in Ghana, especially in terms of exploring perspectives of health workers themselves. There are however, a number of issues that the authors will need to address before consideration for publication. These include the following:  The introduction seems solely based on the availability of donor assistance for healthcare, but it would strengthen the argument if additional literature was included on what are the needs in terms of key healthcare issues in Ghana, and how DAH and donor assistance could help here. There are also very limited references.  An inclusion of a table on the demographics on the included participants would be valuable.  In terms of methods, it is unclear why this study is seen as a cross-sectional study when it is qualitative research.  Including the key informant interview guide in an appendix would be valuable.  The authors are also not clear about if they used any kind of software for data analysis-please confirm.  Have the COREQ guidelines been taken into account for reporting qualitative findings?  The discussion section could be more structured, and it would also be valuable to think critically about the limitations of this study. | Thank you very much for the comments  We have revised the introduction  This has been included as Table 1  Cross-sectional study designs could be used for quantitative or qualitative research – though they are often associated with the former. Since our study collected data for one time point, we consider this as cross-sectional. However, we have opted to use exploratory qualitative study (which also reflects this study), following from comments from the Editor and reviewer.  This has been included in the Appendix  We used Nvivo 12. This was indicated under data analysis section of the methods.  We have reported findings using COREQ guidelines.  We have revised the limitations. |
| 7  8  9  10  11  12  13  14  15  16  17  18  19 | REVIEWER 1  The title has grammatical errors.  ABSTRACT:  The authors should remove “(lower)” from the second sentence in the background, as it is confusing.  The authors only mention Gavi when speaking about global funding agencies. Is there a particular reason for this?  The authors should rephrase the aim to clarify that they are wanting to explore. Is it frontline health workers’ perspectives of the implications of Ghana’s transition to a middle-income country on service delivery?  Are the results summarised from the perspective of frontline health workers? In places, it is not clear what are the perspective of participants and what are perspectives of the authors.  INTRODUCTION:  The authors should reference these sentences: “The shift of a country from low-income to a higher income bracket (such as middle-income) give development partners the indication that a country is in a better position to finance its activities, hence, a reduction in DAH. Thus, countries seeing significant economic growth are no longer eligible to receive assistance, being perceived as qualified to self-finance their health programs.”  Last paragraph: The authors should provide a stronger rationale for why the perspectives of frontline health workers are important and who can benefit from this study. It is also unclear what information on donor aid transition is inadequate, compared to what, and how it can help.  METHODS:  Study design: The authors should phrase the study design as an exploratory (or descriptive) qualitative study, rather than using the term “cross-sectional”. It is also advisable for the authors to be consistent In describing the aim in this study in abstract and main text, and therefore use one term throughout, either “perspectives” of “perceptions”.  Study setting: How were the three hospitals selected and why?  Sampling technique: Can the authors clarify whether they considered which departments health workers worked in and how this could have influenced their perspectives?  Data analysis: Were the interview recording transcribed verbatim? Were any principles of scientific rigour applied, e.g. peer debriefing, audit trails etc.?  RESULTS:  The results are well-described, with supporting quotes.  CONCLUSION:  Do the authors have any recommendations for future research? | Thank you for this comment, but we cannot identify any grammatical errors with the title.  This has been corrected.  We have rephrased the sentence to show that we only cite Gavi as one example of global funding mechanisms that countries graduate from as they move to a middle-income status.  We have rephrased the sentence.  The results are summarized from the perspective of participants.  We have rephrased the sentences and provided citations.  This paragraph has been revised.  This has been addressed under comment 4 above. We have changed all “perception” to “perspective”, consistent throughout the manuscript.  The hospitals were selected purposively, this has been indicated under the study setting. There are key hospitals in Accra that provide services to patients including ART clinics that are mainly funded with external resources (Global Fund, etc)  Within each hospitals, we focused on public health departments, which are often financed from external sources (through public health programmes). This has been explained under sampling techniques.  Interview recordings were transcribed verbatim. Two members of the team reviewed transcripts to ensure that they were consistent. This has been included in the data analysis section of the methods.  Thank you for the compliments.  we have included these in the conclusion. |
