# Supplementary material for: Perspectives of frontline health workers on transition from development assistance for health in Ghana: A qualitative study
Source: PLOS Glob Public Health. 2022 Apr 22;2(4):e0000093. doi: 10.1371/journal.pgph.0000093 (PMC10021137; doi:10.1371/journal.pgph.0000093)
Supplement: Attachment — Submitted filename: Response to comments_Jan_22.docx [file pgph.0000093.s004.docx]

| **S/N** | **COMMENT** | **RESPONSE TO REVIEWER COMMENTS** |
| --- | --- | --- |
| 1  2  3  4  5 | **Comments from Editor**  Introduction: -the changes recommended by the editor have not been made-please see original comments. The literature is very much still based on the DAH only, and no real description of heath issues faced in the country are evident.  COREQ guidelines-the authors say that these have been included. Can more specific information be provided in the responses to confirm this, an if helpful the guidelines checklist can be included as an appendix.  -Please can you add more detail on how telephonic consent was obtained.  Discussion -add the year published to Mao et al  -Limitations: thank you for adding some information here, but this is very brief. Think about the effect on qualitative work that telephonic interviews may have had, and the fact that the data is not generalisable. | Thank you very much for the comments. We have addressed all changes requested by the reviewers. We have also revised the introduction to include information on health outcomes.  Under “Data analysis”, we have included the following sentence: “Reporting of the findings of this study follows the Consolidated criteria for reporting qualitative research (COREQ).” The COREX has been attached as supplementary material  This has been explained in a bit more detain under “ethical considerations”  For each line of the discussion where Mao et al was indicated, we have included the in-text citation showing the year of publication  We have addressed these comments under the limitations sections. |
