# Supplementary material for: Perspectives of frontline health workers on transition from development assistance for health in Ghana: A qualitative study
Source: PLOS Glob Public Health. 2022 Apr 22;2(4):e0000093. doi: 10.1371/journal.pgph.0000093 (PMC10021137; doi:10.1371/journal.pgph.0000093)
Supplement: Attachment — Submitted filename: Response to comments_Feb_22.docx [file pgph.0000093.s005.docx]

| **S/N** | **COMMENT** | **RESPONSE TO REVIEWER COMMENTS** |
| --- | --- | --- |
| 1  2  3 | I would like to thank the authors for the revisions that they have made to this manuscript, which have improved the article. However, there are still three minor areas that I would like to see some changes on, as listed below:  1) COREQ guidelines: please ensure that these match up to what is in the text of your article. There are items under relationship with participants that are marked as n/a in the guidelines, but then are explained in the text. Please rectify this.  2) informed consent: thank you for revising this. However, it is still not completely clear what happened-were participants sent a whatsapp just to read, or was there a way that they could respond in any way. if verbal consent was obtained on the phone, how was this done-did the researcher still sign a hard copy of the document? Some more detail is needed here.  3)limitations: this section is more explanatory now, but I would just like to see an extra sentence on the generalisability of the study findings-to what? other frontline health workers? in Ghana or other countries? in rural vs more urban settings? this really just requires a bit more explanation. | Thank you  The COREQ guidelines have been reviewed and revised where appropriate.  As indicated under ethical considerations, consent was sent to participants via Whatsapp, but they were not asked to respond back via same medium. Before each interview via telephone, participants were reminded of the consent, and they voluntarily gave verbal consent. The interviewer did not sign hard copy form on their behalf, as there is evidence of the verbal consent – the audiotapes.  We have explained this a some detail under the limitations |
